# Quantum Physics Perspective on Electromagnetic and Quantum Fields Inside the Brain

**DOI:** 10.21315/mjms2020.27.1.1

**Published:** 2020-02-27

**Authors:** Zamzuri Idris

**Affiliations:** Brain Behaviour Cluster and Department of Neurosciences, School of Medical Sciences, Universiti Sains Malaysia, Kelantan, Malaysia

**Keywords:** electromagnetic field, quantum field, brain, universe, wave, energy, mind, psychosis

## Abstract

Brain energy is associated commonly with electrochemical type of energy. This energy is displayed in the form of electromagnetic waves or better known as brainwaves. This concept is a classical concept (Newtonian) in which the studied object, that is the brain is viewed as a large anatomical object with its functional brainwaves. Another concept which incorporates quantum principles in it can also be used to study the brain. This perspective viewing the brain as purely waves, including its anatomical substrate. Thus, there are two types of energy or field exist in our brain: electromagnetic and quantum fields. Electromagnetic field is thought as dominant energy in purely motor and sensory inputs to our brain, whilst quantum field or energy is perceived as more influential in brain cognitions. The reason for this notion lies in its features which is diffused, non-deterministic, varied, complex and oneness.

## Quantum Physics for the Brain

Quantum physics is the branch of physics that deals with tiny objects and quantisation (packet of energy or interaction) of various entities. In contrast to classical Newtonian physics which deals with large objects, quantum physics or mechanics is a science of small scale objects such as atom and subatomic particles. In general, central elements of quantum physics are: i) particle-wave duality for quantum entity such as elementary particles, and even for compound particles, for instances, atoms and molecules; ii) quantum entanglement. It can be defined as a phenomenon in which the quantum states of two or more objects have to be described with reference to each other, even though the individual objects may be spatially separated; iii) coherence and decoherence. Coherence is referred to waves that have a constant phase difference, same frequency, or same waveform morphology, whilst decoherence means interference is present; iv) superposition. It is a complex property of a wave. In a wave, there could be many other smaller waves; v) quantum tunneling. It is a phenomenon where a quantum particle passes through a barrier; vi) quantum uncertainty. It is also known as Heisenberg’s uncertainty principle. It states that the more precisely the position of some particle is determined, the less precisely its velocity can be known, and vice versa (1). Thus, quantum physics is seen as dealing with ambiguity in the physical world.

Based upon the first principle, human brain can be viewed entirely as either in particle or in wave form. The particle perspective portrays brain in anatomical form, whilst the wave perspective depicts the brain in wave form (2, 3). Waves of the brain can be classified further into two main entities: i) brainwaves that commonly detected or studied using electroencephalography (EEG) or magnetoencephalography (MEG) and is based on electromagnetic principles; and ii) waves perspective of brain anatomical particles. The first waves or brainwaves can be named as electric waves with energy or field in it—thus stated here as electromagnetic field (EMF) and for the second waves, quantum waves or quantum field (QF) is used. Therefore, brain can be viewed as either i) anatomical brain with brainwaves (classical) or ii) all are waves with onefield or energy, but this field can be divided further into EMF (large object physics) and QF (small object physics). In this editorial commentary, the author describes briefly these two concepts of brain fields and invite readers to think of quantum physics as a science that not only capable in describing the behaviour of subatomic particles, but also behaviour of people (people mind).

## Electromagnetic and Quantum Fields Inside the Brain

**Figure 1:**
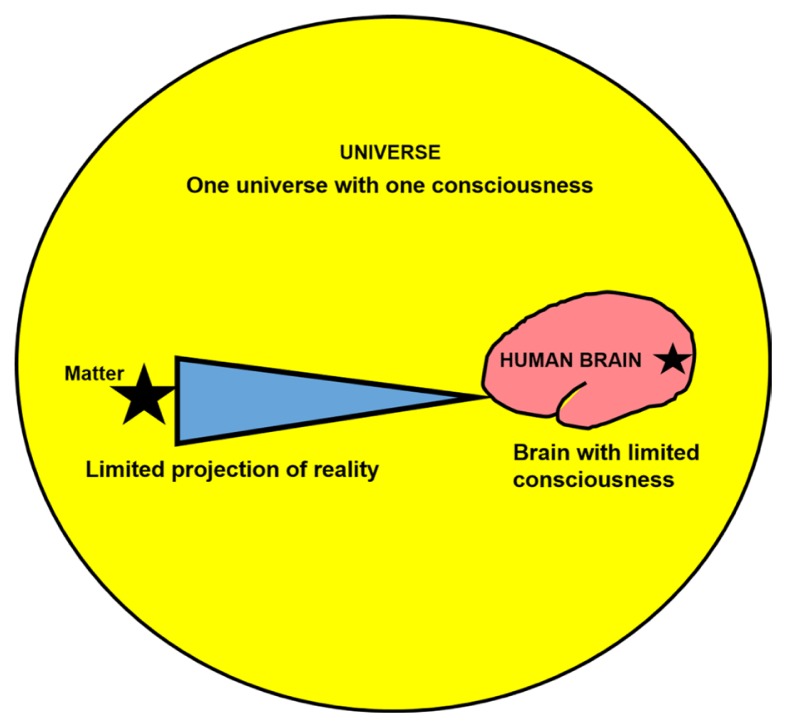
Limited consciousness for the brain and limited projection for the universe form a principle for the collapse of wave function of the particle. Brain and universe are permeated by quantum field, whilst electromagnetic field inside the brain arises from a discrete or limited projection from our universe

A physiological principle states a neuron communicates with each other by using electrical signals. The electrical signal called action potential travels along the axon and triggers neurotransmitter at the synapse, hence further electrical signal can be passed to other neurons. With electrical signal, there is always a simultaneous presence of magnetic field. Thus, this type of communication is known as EMF communication (4–6). On the contrary the QF type of communication considers all brain elements are waves, thus the energy is still wavy (ups and downs) and perhaps in diffused pattern with more complex networks. In this perspective, EMF of the brain is viewed as arising from: i) a projected stimuli outside the brain such as our five senses of stimuli—seeing, hearing, touching, tasting, and smelling; or ii) the brain itself such as in virtual reality, dreaming and hypnosis (without external stimuli); and iii) non-cognition such as pure motor movements. On the other hand, brain QF is viewed as onefield or wholeness or oneness with our universe. Thus, it is commonly regarded as having one consciousness (7, 8). With this understanding, consciousness concept in quantum realm is not restricted only to human brain. In other words, we may say QF permeates whole of our universe. The quantum entity that suits this permeating energy concept is the light, whilst the non-quantum entity (Newtonian physics) that suits the focus or limited projection is electricity. Hence, EMF has electric feature whilst QF has light feature. This is summarised in Figure 1 and Table 1.

The two aforementioned concepts (i.e. projected stimuli or internally arising stimuli for EMF and brain as part of one consciousness) unintentionally introduce a ‘limited’ principle for both our universe and brain. For our universe, the projected stimuli is a limited stimuli from only certain aspect, area or dimension of our universe; and for the brain, the limited principle is applied for the consciousness—our brain is part of one consciousness or has limited consciousness. With these ‘limitation principle of our universe and brain’, our brain (3D-vision) cannot see wave function of a particle or atom, we can only see them in particle, atom, molecule, matter or object form. In other words, we may say partial consciousness that we have, collapsing the wave function of a particle and thus limited our perception to only three-dimensional vision (particle, atom, molecule, matter).

## EMF and QF in Relation to Medicine

In reference to Table 1, brain EMF is based on electric signal that has pilot or directional high frequency, short wavelength waves that are locatable or can be determined using few stimuli or trial with noted large evoked electrical (or magnetic) response. Thus, it seems to cover our five basic senses with simpler brain networks. Conversely, light (also a type of electromagnetic spectrum) is regarded as main entity for brain QF. It is diffused or non-directional lower frequency and longer wavelength waves that are unlocatable or non-deterministic or varied. Other features of QF are more symmetry (light feature) and having more complex networks. Thus, QF may play bigger role not in our five common senses, but more in our brain cognitions. With these features (varied, complex, diffused), neuroplasticity is thought to happen more likely in cognitive (language, emotion, memory, attention etc.) than in our five-sense or motor impairments.

With the aforementioned reasons, patients who are suffering from motor, sensory or cognitive impairments obviously require EEG or/and MEG in order to come out with better diagnosis or better knowing the extent of impairment. Those suffering from psychiatric disorders or psychosis spectrum disorders, QF is probably a better energy that should be studied in them. It is because of oneness or wholeness concept for QF; any fragmentation in this onefield (loss from reality) likely causes psychotic-like manifestation (9, 10). Noteworthy, more symmetry and lower frequency waves features of QF may be utilised in making diagnosis and monitoring for psychiatric disorders. In relation to this understanding, one may treat cognitively impaired or psychotic patient by using a more diffused, smaller and multiple electrodes (toothbrush-like electrodes) implanted at certain cognitive or psychosis brain networks.

## Conclusion

Universe and brain are considered as two most complicated entities with obvious links exist between them. One of those links is ‘limitation principle’ for both. The energy in our brain is thought as pairing, with obvious EMF and more hidden QF energy. QF is thought as a permeating background energy for our brain and universe, while EMF is more focus-, limited- and projected-like brain energy. Greater understanding in QF may open new ways on how to treat some medical disorders, particularly ones that related to cognitive impairment and psychiatry.

## Figures and Tables

**Table 1:** General features of brain EMF and brain QF

	Feature	EMF	QF
1	Wave pattern	Presence of pilot/directional wave	Diffused waves
2	Wave characteristics	High frequency wave	Low frequency wave
3	Wavelength	Short wavelength	Long wavelength
4	Quantum concept	Deterministic (locatable)	Non-deterministic (unlocatable, varied)
5	Dimension	High dimension (electric)	Low dimension (light)
6	Physics concept	Bohmian mechanics	Quantum mechanics
7	Brain network	Simple network (few nodes)	Complex network (many nodes/varied)
8	Symmetry	More asymmetry	More symmetry
9	Evoked response	Large evoked response with few stimuli (few trials/tests)	Smaller evoked response and need higher amount/number of stimuli (multiple trials/tests)
10	Neuroplasticity	Less	More
11	Wholeness/Oneness/Onefield concept	No (it is a projected/limited field)	Yes (spreading or permeating whole of universe/field)
12	Related to psychiatry	Less relevant	More relevant, because this QF is related to wholeness or reality or one consciousness concept
13	The way to alter the network	Focus few electrode [deep brain stimulation (DBS) like electrode for Parkinson etc.]	Smaller and multiple electrodes (toothbrush like electrodes)
14	The way to alter the network using frequency	High frequency is preferred in most cases (inhibition)	Low (stimulation) and high frequency stimulation depends on clinical manifestations

	**Brain function**	**Combination of EMF and QF (two brain fields/energy)**	

A	Brain function (motor, sensory, vision, sound, touch) and its impairment	Non-cognitive impairment such as stroke affecting motor, sensory, vision, sound, touch EMF is more affected than QF Measureable Associated with degree of impairment	
B	Brain function (language, emotion, memory, attention, planning etc.) and its impairment	Cognitive impairment for language, emotion, memory, attention, planning etc. QF is affected significantly together with EMF Measureable Associated with degree of impairment	
C	Brain function and psychosis	Psychotic manifestations such as auditory or visual hallucination, thought insertion, delusions etc. QF is more affected than EMF Yes or No (presence or not) (not associated with degree)	
